# Acknowledgment to the Reviewers of *Current Oncology* in 2022

**DOI:** 10.3390/curroncol30020099

**Published:** 2023-01-17

**Authors:** Current Oncology Editorial Office

**Affiliations:** MDPI AG, St. Alban-Anlage 66, 4052 Basel, Switzerland

High-quality academic publishing is built on rigorous peer review. *Current Oncology* was able to uphold its high standards for published papers due to the outstanding efforts of our reviewers. Thanks to the efforts of our reviewers in 2022, the median time to first decision was 20 days and the median time to publication was 42 days. Regardless of whether the articles they examined were ultimately published, the editors would like to express their appreciation and thank the following reviewers for the time and dedication that they have shown *Current Oncology:*
Aaron C. TanLawrence Frank PaszatAbbas MardaniLayth Mula HussainAbdulhameed Al-GhabkariLazaros TzelvesAbdulla Al-RashdanLeah M. PyterAbdulqadir J. NashwanLei GaoAbel JosephLein‐Ray MoAbhilasha SinhaLeonard Christopher SchmeelAbhishek KumarLeonardo BencivengaAbid UllahLeonardo CentonzeAbigail Morales-SánchezLeslie J. HinyardAda PopoloLetizia DeantonioAdam Edward LangLi-Min WuAdelaida AvinoLiang WangAditya Kumar GuptaLianne J. BarniehAditya WaghLijen WangAdrian Elmi-TeranderLiliana MontellaAdriana Aguilar-LemarroyLin WuAdriana GrigorașLinda Katherine JonesAdrienne L. WatsonLinlin GuoAfshan Fathima NawasLiverana LaurettiAgnieszka Jagiełło-GruszfeldLloyd MackAgnieszka MicekLong Chiau MingAgnieszka PotęgaLoredana MarcuAh YusufLorena Garcia Del RioAhmed Abu-AwwadLorenzo BelluominiAhmed HamaïLorenzo FaggioniAhmed MoustafaLoris De CeccoAkihiro KuwahataLourdes Cortes-DericksAkiyoshi OginoLovorka BrajkovićAkshay PandeyLu Ping TanAlain Antoine MinaLubbers TimAlan KirkLuca BertolacciniAlana RojewskiLuca Di GianfrancescoAlannah M. SmrkeLuca FalzoneAlbert BieteLuca GiacomelliAlbert GrinshpunLuca Lo NigroAlbert OriolLuca OngaroAlberto AntonicelliLuca SteardoAlberto Carmona-BayonasLuca ViganòAlberto LeardiniLucas DelmonicoAlberto MaccariLucia CannellaAlberto RomanoLucia MangoneAlberto SandriLuciana VintiAldo PezzutoLucie M. TurcotteAlejandro Botero-CarvajalLuigi BonavinaAlejandro Herreros-PomaresLuigi LoscoAlejandro Olivares-HernándezLuigi NapolitanoAleksander ŚlusarczykLuís Carlos Lopes-JúniorAleksei TikhonovLuis Exequiel IbarraAlessandra BorgheresiLuis Mariano EstebanAlessandra BosuttiLuis RodrigoAlessandro BaisiLuiz Carlos Conti De FreitasAlessandro D'AmuriLuka FlegarAlessandro MazzottaŁukasz KuncmanAlessandro RizzoŁukasz MasiorAlessandro VittoriŁukasz NawackiAlessia BariŁukasz ZapałaAlessio PaladiniLut TamamAlexander KleinLuz Berenice López-HernándezAlexander TamalunasMaciej JaneczekAlexandra GeusauMaddalena Di SanzoAlexandra O. SokolovaMaeirah AshaieAlexandra Von AuMagdalena Knetki-WróblewskaAlexandre González-RodríguezMahdi SheikhAlexandros LaiosMahesh KasthuriAlexey SuminMahtab SamimiAlexey V. PetukhovMaja Rogić VidakovićAlexis KoskanMajid VahedAlexis UlrichMakkai-Popa Silviu-TiberiuAlfonso BaldiMakoto EndoAli BoolaniMalcolm BrinnAlper SozutekMałgorzata Dorota SkibinskaAlvaro MoralesMałgorzata KlimekAlyson MaharMalgorzata KrawczykAmadeus DobrescuMałgorzata PasekAmanda J. ChaseMalgorzata Trofimiuk-MüldnerAmanda WurzMallorie B. HeneghanAmaniel KefleyesusManabu NatsumedaAmaury DasteManish CharanAmbica ParmarManoj KumarAmelia Maria GamanManon LemondeAmir AshoorzadehMansoor HussainAmirhesam BabajaniManu PrasadAmit K. AroraManuel ValdebranAmit KumarManuela CaroliAmit Kumar JaiswalMara CarsoteAmit RoshanMarc DenisAna DascaluMarc OsterlandAna Isabel PadrãoMarc PocardAnas ShamsiMarcin BalcerzykAnastasios NtavatzikosMarcin BobińskiAndraẑ DovnikMarcin JozwikAndrea AntonuzzoMarcin NicośAndrea BalliniMarcio Alvarez-SilvaAndrea BellieniMarco Antonio Meraz-RíosAndrea CassoniMarco CaricatoAndrea Dell'AmoreMarco ClementiAndrea Di BlasioMarco FoganteAndrea GeorgiouMarco MontilloAndrea Luigi Camillo CarobbioMarco TonelloAndrea MulliriMargaret OttavianoAndrea SambriMargarete SteinerAndrea SpiniMargita PobijakovaAndreas AgathangelidisMaria Alexandra PapadimitriouAndreas C. KiriakopoulosMaria AmbrosioAndreas KriegMaria Chiara BruneseAndreas PolydorouMaria ContaldoAndrei ColitaMaría Dolores Llobat BordesAndrei Mihai MalutanMaría Dolores Ortiz-MasiáAndrej PalaMaria Giulia CangiAndrew DalbyMaria Gomes Da SilvaAndrew J. KobetsMaría Isabel Tovar-GálvezAndrew JinMaría Jesús Alvarez CuberoAndrzej KasperskiMaría José Pérez LacastaAndrzej KurylcioMaria LitwiniukAndrzej NowickiMaria Lucia Hirata KatayamaAndy WilsonMaría M. Morales Suárez-VarelaAngela Dalia RicciMaria MazzoniAngela MastronuzziMaría Ramos-MonserratAngelena CrownMaria Rita BiancoAngelos SikalidisMaria Rosaria RicciardiAnita Kloss-BrandstätterMaria Teresa VietriAnja Kathrin WegeMaria VaccaroAnjali KusumbeMarialuisa LugaresiAnkit ManglaMariangela ManciniAnna AcamporaMarianne TinguelyAnna Andreevna StarshinovaMarie LangeAnna AureliMarie-France SavardAnna CybulskaMariia D. IvanovaAnna DianaMarika QuadriAnna GolebiewskaMarina MencingerAnna KleczkaMarina MichalakiAnna M. E. BruynzeelMarinela BostanAnna Maria FrustaciMarino VeneritoAnna Mucha-MałeckaMario De FeliceAnna StarzyńskaMario MischkulnigAnnalisa MasiMario Perez SayansAnnalisa TramaMarion CortetAnnamaria PorrecaMarisa R. BuchakjianAnne Marie RuppertMarius Gustav BredellAnneli ElmeMariusz DuplagaAnnette B. PfahlbergMark P. LythgoeAntal ZemplényiMark R. SpeicherAnthea C. PetersMarko KavčićAnthony AlbergMarko KorhonenAnthony FieldsMarkus BallAnthony HerbertMarkus QuanteAnthony M. VillanoMarkus SchäferAntoine AdenisMarkus WilhelmiAntonella ArgentieroMarlena GodlewskaAntonella CacchioneMarlon MundtAntonello PintoMarta F. EstradaAntoni SzczepanikMarta OpalińskaAntonia KondouMartha Patricia Gallegos-ArreolaAntonio AmmendoliaMartin BakhtAntonio BarileMartin JanickoAntónio CaleiroMartin MischAntonio CicioneMartin MynarekAntonio Claudio TrainoMartin StimpfelAntonio FacciorussoMartina AlvarezAntonio G. SolimandoMartina KirchnerAntonio Giovanni SolimandoMartina KleberAntonio Jesús Molina FernándezMartina SalákováAntonio MacciòMartina T. MoglAntonio MazzellaMary Beth MadonnaAntonio SchiattarellaMary Jane EsplenAnudishi TyagiMasahiro TajikaAo ZhangMasahito ShimojoApostolos PapachristosMasao MiyagawaArash NavranMasashi KanaiArcangelo SebastianelliMassimiliano De PaolisArmando PatrizioMassimo BergerArnaldo StanzioneMassimo CordaroArnaud AlvesMatei BratuArnaud PagesMateusz Jacek SpałekArndt StahlerMateusz JankowskiArne WestgaardMathieu ChocryArpita ChatterjeeMathieu LuyckxArpita RaiMathilde C. M. KouwenhovenArtur LemińskiMatteo GhilliArturo BonomettiMatteo RenzulliArunima SikdarMatteo RiccòArya AshokMatthew A. SteligaAshish ManneMatthew ChanAshokkumar ThirunavukkarasuMatthew DixonAthanasios T. PapadasMatthew G. K. BeneschAtsushi SanoMatthew H. RigbyAttila PatocsMatthew PainschabAtul BatraMatthias ScheweAudrius DulskasMattia Falchetto OstiAvinash WaghrayMattia FrascioAvram DenburgMattia RiefoloAyanthi A. WijewardeneMaura MassiminoAydin EresenMaurizio MartiniAyrat U. ZiganshinMauro BorzioAyşe Arıkan DönmezMauro Giuseppe MastropasquaAyse Günes-BayirMaximilian ChristopeitAyslan Castro BrantMay Ee PngBalachandar VellingiriMayo TsuboiBálint TamaskovicsMcKayla J. RiggsBarbara DziegielewskaMd Islam SharifulBárbara Paranhos CoelhoMeden F. Isaac-LamBarbara VerroMegan BeetchBartek SzostakowskiMegan DelisleBartosz ChmielowskiMeifen ZhangBartosz MałkiewiczMeinolf SuttorpBaudolino MussaMelissa D. CantleyBeatrice AlbanesiMelissa L. HillBehrus PuladiMeng-Che HsiehBen MorrisMichael A. JacksonBenjamin AnsaMichael DeanBenjamin C. P. LeeMichael DevaneBeppe CalòMichael GreenBernard MalavaudMichael GrunertBernd MetznerMichael J. KaplanBethany Hipple WaltersMichael J. NathensonBethany N. SmithMichael JonesBhawna DevMichael KolinskyBhupesh ThakurMichael L. CherBiagio BaroneMichael LeeBilqees BanoMichael LingenfelderBinu Kandathilparambil SasiMichael RückertBita GeramizadehMichael W. BishopBjörn NashanMichail DiakosavvasBlanca Lumbreras LacarraMichal BraunBlazen MarijicMichał KulusBo MussmannMichał P. WasilewiczBogdan Silviu UngureanuMichał ZarobkiewiczBogdan SoceaMichalis LiontosBonnie Gould-RothbergMichel Van Den HeuvelBonnie LeungMichele FinottiBora LimMichele FioreBorek SehnalMichele SicaBouthaina DabajaMichelle GhertBradley EfronMidori Filiz NishimuraBrandi N. ReevesMiglė BacevičienėBrandon M. HuffmanMiguel A. OrtegaBranko KolaricMiguel Ángel Canales AlbendeaBrian D. KellyMihaela BacalumBrian GodmanMihai CapilnaBrian O'RourkeMihai Dorin VartolomeiBrieuc SautoisMihai Stefan MuresanBrinda BalasubramanianMihai-Stelian RusuBrittany MurphyMihir ShettyBruce M. BomanMiho KawaidaBruno José Nievas SorianoMikhail SinkinBruno KastlerMikhila MahendraC. M. Sabbir AhmedMilad MoloudizargariCaitlin TresslerMin Soon ChoCamelia CoadaMinakshi GandhiCameron K. TebbiMine BekarCandice JohnstoneMing-Hsin YangCapucine BaldiniMing-Hui YangCarl D. MalchoffMing-Ming TsaiCarlo IraceMingyi ChenCarlo RonsiniMinoru ShigekawaCarlos Eduardo Fonseca-AlvesMiodrag DragojCarlos Felipe Rengifo RodasMirco MasiCarlos GalanMiri Sklair-LevyCarlos Martin LlorenteMiriam BoppCarlos ViegasMirjan M. NadrljanskiCarmen Guillén-PonceMiroslav SamaržijaCarmen NeamtuMirza PojskicCarmine CitarelliMischa De RidderCarmine ConteMishall Al-ZubaidieCarole HelisseyMitchell SabloffCaroline Aquino Moreira-NunesMitja MitrovicCaroline MarianoMohamed HassanCarolyn FreemanMohammad A. SaadCarolyn HeckmanMohammad SouriCary PresantMohammad Usman AhmadCatherine LabbéMohammed Abu El-HamdCeleste CagnazzoMohammed AlaswadCelestia Tia HiganoMohsen IbrahimCelina AngMohsen YazdanianCemil ColakMojtaba MokariCéu CostaMomcilo JankovicChakraborty SohiniMona VintilaChandrakanta MahantyMonica BalzarottiChang-I ChenMonica Livia GheorghiuChangchuan JiangMonika Peshevska-SekulovskaCharat ThongprayoonMonika RucinskaCharles DietrichMonika SobočanCharles F. StreckfusMonika SzeligaCharles Van PraetMonish Ram MakenaChe-Hsueh YangMoon Young KimChen Guo KerMoreno ZanardoChen ZhaoMorteza Fallah KarkanCheng-Hsu WangMotaz HamedChengfei JiangMototsugu OyaCheryl BeselerMotoyuki UmekawaChi Chun WongMuhammad Khalid Khan NiaziChi Fu Jeffrey YangMuhammad N. MahmoodChiao-En WuMukai Chimutengwende-GordonChiara CortiMumtaz AnwarChiara Maria MazzantiMustafa S. AschaChien-Chih ChenMyung-Gwan KimChien-Feng LiNa WangChin-An YangNadeem BilaniChin-Man WangNadine S. AguileraChing-Feng WuNancy BaxterChloe FletcherNandakumar KrishnadasChris LabakiNannan LuChristian ScheiweNao KakizawaChristina Oetzmann Von SochaczewskiNarendar DudhipalaChristine KochNariman NezamiChristine MaheuNatália AraújoChristopher M. BoothNatércia ConceiçãoChristopher Paul VennerNathalie AndréChristopher PykeNathalie S. GuedjChristos ChouaidNathan H. ParkerChristos MikropoulosNatsuo TomitaCindy H. ChauNavid RazmjooyCiro ImbimboNazanin MajdClare SchillingNazario FoschiClaudia CocchiNebojsa S. ManojlovicClaudia MehedintuNeetika NathClaudio AndreettiNetta BenturClaudio Bovolenta MurtaNeville HackerClaudio LucchiariNguyen Minh DucClyde MartinNibedita PatelColin H. QuinnNicholas FidelmanColm O'RourkeNicholas P. RowellConcepción Perez-HernandezNick KouttabConcetta PirontiNico TeskeCoonoor ChandrasekarNicola GentiliCorrado CasliniNicola MontemurroCorrado TinterriNicola PavanCosimo SpertiNicolae GicaCraig R. UnderhillNicolas De L'EscalopierCrismita DmelloNicolas SayeghCristi TartaNicolò BrandiCristian DelceaNikita NikitaCristiana CarnitiNikolaos MachairasCristiana Lo NigroNikolaos V. MichalopoulosCristiana VidaliNiluja ThiruthaneeswaranCristina M. FaillaNina RembiałkowskaCristina TrovatoNing JinCyrus MotamedNorihiko NaritaD. S. PrabakaranNoriko KishiDa-Liang OuNorma G. Padilla-EguiluzDa-Yong HouNorman R. WilliamsDagmar PavluNuha A. El-SharifDagmara Otto-ŚlusarczykOctavian AndronicDagmara Szmajda-KrygierOfer BaranovitchDahang YuOlarik SurintaDamiani GiovanniOlav YriDamiano CarusoOle PetersenDamir GrebićOle SolheimDan Cristian RaduOlexiy AseyevDana CucuOlga BureninaDane KrtinicOlga NigroDaniel BreadnerOlga Y. KorolkovaDaniel EigerOliver J. OttDaniel LindsayOlivier MolinierDaniel M. SciubbaOra NakashDaniel Marrero-RodríguezOreste GalloDaniel Patiño-GarcíaOrestis LyrosDaniel S. SitarOriela RustemiDaniela CalinaOrit Kaidar-PersonDaniela Cornelia LazărOrlando PariseDaniela DamianiOscar ArrietaDaniela DoegeOsman ÖcalDaniela SuzukiOthman AkhtarDaniele DerudasOttavia D'OriaDaniele ScartoniOuti KuittinenDaniele Ugo TariOxana DobrovinskayaDanilo De NovellisOxana SerovaDanushka SeneviratnePäivi M. LähteenmäkiDanuta Gąsior-PerczakPamayla DarbyshireDario FerreroPamela Bond CassidyDariush IraniPanagiotis G. HalvatsiotisDariusz KrokPanagiotis LainasDavid BerbrayerPanagiotis TsagozisDavid E. DawePandurang KolekarDavid FornerPaola De LucaDavid Ilan SusterPaolo OrsariaDavid J. EatonPaolo SpinnatoDavid KriebelPaolo VeronesiDavid MathieuParaskevas LybérisDavid RobergePascal LambertDavid S. AllanPaško KonjevodaDavid S. SiegelPasquale AvellaDavid StewartPasquale CianciDavide DonnerPasquale PisapiaDavide LeardiniPatrice MathevetDavide RadicePatrice RollDebjani PalPatricia CantoDebmalya BarhPatricia FitzpatrickDebora CapelliPatrick HerrDeborah J. VestalPatrizia DorangricchiaDeepanjan PaulPaul CottuDeependra Kumar SinghPaul Stephen RavaDeepesh GuptaPaul Van HoutteDeepti BehlPaul Wheatley-PriceDeepti ChopraPaula BeneveneDenis QuerleuPaula DoboszDenise Shuk Ting CheungPaula GordonDenise ToscaniPaula K. BaldwinDervla KellyPaula OlaizolaDespina BazouPaula Poikonen-SakselaDhan ShresthaPaulina CeglaDhananjay YadavPavan Kumar DhanyamrajuDi ZhangPavol DubinskýDiana MartinsPaweł DerlatkaDiana N. IonescuPaweł DomagałaDiane Von AhPaweł SobczukDiesch TamaraPedro Villarejo-CamposDieter BroeringPeiwei GongDimitri AnzelliniPeng WeiDimitrios KotsirisPengze YanDimitrios PapandreouPenny K. SneedDimitrios ParaskevopoulosPer HydbringDimitrios TzachanisPer Olof LundgrenDina Di GiacomoPernille HolmagerDinesh Chandra DovalPetar OzretićDionysios C. VorosPeter ArgentaDirk J. Van LeeuwenPeter HauserDirk SpitzerPeter HollanderDivanshu ShuklaPeter M. AndersonDivya GopinathPeter Stephen VoslerDmitrii ShekPhilippe JoubertDoerte WichmannPhilippe SoyerDomenico AlbanoPhillip S. BlanchetteDomenico FuscoPia Z. Pace-AsciakDomenico MaisanoPier Adelchi RuffiniDomenico MotolaPier Paolo PiccalugaDomenico SolariPierfrancesco FrancoDomenico TripodiPierfranco ConteDominik DłuskiPiero FregattiDominik RadzkiPietro BertoglioDominik SaulPietro CarotenutoDominik SchneiderPietro MorroneDominique ModrowskiPing ZouDominique Valteau-CouanetPiotr DzięgielDon Thiwanka WijeratnePiotr MorasiewiczDongdong CaoPiotr Oskar CzechowskiDoreen EzeifePiotr RichterDorian Yarih Garcia-OrtegaPlacido BruzzanitiDorry BollPo-Yuan ChenDouglas Allan StewartPorfyrios KorompelisDragos MedianPrabakaran NagarajanDulani MeedeniyaPrafull GhatageDumitrescu CatalinPranabananda DuttaEason HildrethPranshu SahgalEd SchmidtPrashanth Kumar Bhusetty NageshEduardo Gutiérrez-AbejónPratiti BhadraEdward Christopher DeePraveen Kumar NeeliEdy IppolitoPreetish Kadur Lakshminarasimha MurthyEelco De BreePriya RamanathanEetu HeerväPriyanka ShawEiji HishinumaPunam Prasad BhadaniEirini KatodritouPuneetpal SinghElaine Souza-FagundesQing ChangElaine Yee Lin ChungQuan ZhouElectra D. PaskettQuincy ChuElena Adelina TomaRachel L. WashburnElena BernadRadu AlbulescuElena QuaglinoRafael Camacho-CarranzaElena RizaRafael Ríos-TamayoEleni TimotheadouRafał GerymskiEleonora BinattiRaghunadharao DigumartiElie El HelouRaghuveera GoelElin BørøsundRahul KumarElisa BelluzziRahul MannanElisa SconfienzaRajeev PandeyElisabetta RazzuoliRajeev SinglaElizabeth C. PenickRaluca GrigoreElsemieke I. PlasmeijerRamcharan Singh AngomElżbieta CiporaRamesh KumarEman ToraihRamon CleriesEmanuela FogliaRamona BongelliEmanuele D'AmicoRan XuEmi DikaRaquel Guillamat PratsEmiliano DallaRaquel SebioEmilie MamessierRashmi MaddaEmilio EstebanRavi RamjeesinghEmily GordonRavindra DeshpandeEmina SherRavindran Caspa GokulanEmma D'IppolitoRaymond Hugo HendersonEmma MontellaRazvan BardanEmma ThamRăzvan-Ionuț PopescuEmmanouil GiorgakisRebecca E. HoedemaEmmanouil Ioannis KapetanakisRebecca HarrisonEnrico MarinelliRebecca MercerEnrico OddoneRedi RahmaniEnxiang ZhangReena Vohra SainiErcole MazzeoReich WaldemarEric LinRenata De Almeida CoudryEric Suero-MolinaRenata SoliminiErin M. MobleyRenato GalzioErin MorganRenato GondarErshuai ZhangRené HägerlingErtuğrul ŞahinRenelle MeyersErwin BrosensRenzo ManaraEshan KhanResi PucciEsther Oomen-De HoopRiccardo SartorisEttore BidoliRichard L. BennettEuan WalpoleRikio SuzukiEugenia AllegraRima KregždytėEugenio BrunocillaRishi Man ChughEuishin Edmund KimRitesh KumarEun Jeong ParkRobert L. GiuntoliEunyoung KimRoberta GelminiEva Krieghoff-HenningRoberta GonnellaEva LietoRoberto Augusto Ferriz-MartínezEvangelia KoukakiRoberto CasconeEvangelia StalikaRoberto ChimenzEvangelos KoustasRoberto CuttanoEve PécheurRoberto La RoccaEvert F. S. Van VelsenRobyn MacfarlaneEvgenii ShumilovRodica Maricela AnghelEvgeny KlyuchnikovRodrigo ValenzuelaEwa KocotRogelio González-GonzálezEwa KoscielniakRoger Olofsson BaggeEylem Kulkoyluoglu CotulRoger OveEzekiel WeisRoland MertenFabian WeykampRoman GoněcFabiana NapolitanoRonald A. LubetFabio BozziRonald B. BrownFabio CampanellaRosa Maria GerardiFabio CarboneRossella BrunoFabio GasparriRossella LianiFabio TirottaRossella SgarzaniFabiola PaiarRoubini ZakopoulouFabrice DarcheRowland HanFarzad PakdelRoxana PopescuFarzaneh KordbachehRóża DzierżakFausto MaffiniRui DangFederica FrezzatoRui Farinha FarinhaFederica MagagnoliRui VitorinoFederico CaobelliRuitu LyuFederico DehoRuiying ZhaoFederico FerrariRupsa BhattacharjeeFederico PozzoRusha PatelFelipe Andrés Cordero Da LuzRussell KabirFelipe José AidarRut Lucas-DomínguezFelix BraunRute SantosFelix HüttnerRutugandha ParanjpeFemke JansenRyan SukFerdinando AgrestaSabela ParadelaFernando GuimarãesSabrina Rahman ArchieFernando Vázquez-AlonsoSachio FushidaFilippo Tommaso GallinaSachit AnandFilippo TorrisiSagar SohoniFlavio AndrescianiSaima ImranFlora N. BalievaSally LauFlorence Mei Fung WongSalman KimiagariFlorian GebauerSalvatore CocuzzaFlorian GesslerSalvatore SorrentiFlorin Ioan ElecSalvatrice MancusoFoteinos-Ioannis DimitrakopoulosSamantha DicuonzoFouquet GuillemetteSamantha J. MayoFranca FagioliSamantha PollardFranca MelfiSambit Kumar MohantyFrancesca B. FalzaranoSameh GehaFrancesca ColoneseSami Abd ElwahabFrancesca De FeliceSami AkbulutFrancesca FerreSamina AlamFrancesca MartiniSamuel A. KareffFrancesca RicciSamuel T. OrangeFrancesca RossiSan Ming WangFrancesca SanguedolceSanda Cristina OanceaFrancesco BennardoSandi Baressi ŠegotaFrancesco BussuSanjay GuptaFrancesco FelicettiSanjeev GuptaFrancesco GallettiSanna IivanainenFrancesco LeoSara Colomer-LahigueraFrancesco MaffìaSara SchonfeldFrancesco MoraSarika GuptaFrancesco RestelliSasha LupichukFrancesco VoltaSastre-Garau XavierFrancisco PerezSathya Baarathi Shanthi RavichandranFrancisco Reyes-SantiasSatoru MiuraFrancisco Sánchez‐BuenoSatoru MunakataFranco Alchiede SimonatoSaulius CicènasFrancois DionneSavio Domenico PandolfoFranz Villarroel-EspíndolaSavvas FrangosFranz ZehentmayrSavvas LampridisFranziska HerbstSawsan ZaitoneFrédéric MarchalSayantani Sarkar BhattacharyaFrederic W. GrannisSayed Aliul Hasan AbdiFu-Shing LiuSayed K. GodaFu-Zong WuSazan RasulFulden Ulucan KarnakScarfì FedericaFulvio MassaroSchroder SattarFumitaka KogaSeamus O'ReillyFumiyoshi FujishimaSebastian J. SchoberGábor SomlyaiSebastian TorkeGabriel KacsoSebastián Valverde-MartínezGabriela AniteiSebastian YuGabriele TudertiSebastien TaurinGaetano RiemmaSeiichi YoshimotoGaia BarisioneSelami Aykut TemizGang ChenSema K. AydedeGąsowska-Bajger BeataSenija EminovićGatot PurwotoSenshuang ZhengGaurav JoshiSerdar SaritasGavin StuartSerena BertozziGaya Punnia-MoorthySerena DantiGeetanjali Dabral DattaSergey G. AfanasyevGelan AyanaSergey KulemzinGeoffrey KuSergio AlfieriGeorg Martin HaagSergio CortelazzoGeorge Calin DindeleganSergio OcchipintiGeorge DranitsarisSergio Piñeiro-HermidaGeorge ZanazziSerguei KozlovGeorgia KarpathiouSeung-Ho YangGeorgios AndroutsopoulosSevinci PopGeorgios D. MichailShah-Hwa ChouGeorgios LaliotisShahab UddinGeorgios S. MarkopoulosShahenaz NajjarGerardo CazzatoShahid AhmedGerardo MusuracaShaimaa HusseinGermana De NucciShaohua LuGérôme BohelaySharad AwasthiGiacomo Maria PirolaSharlene GillGiacomo SpinatoSharmila FagooneeGian Piero TurchiShazid Md. SharkerGiancarlo PecorariShebli AtrashGianluca FeriniShelly R. McFarlaneGianluca SpenaSherif El‐MasryGiedrius BarauskasSherry Yun WangGinés HernándezShiela AppavooGiorgio BoganiShigao HuangGiorgio MustacchiShigeki YamaguchGiovana TorrezanShigenaga MatsuiGiovanni Codacci-PisanelliShigeo OhbaGiovanni Maria IannantuonoShih-Bin SuGiovanni PalleschiShihlung ChenGiovanni PapaShin NishioGiovanni ScarzelloShin-Hoo ParkGirijesh Kumar PatelShinichi KinamiGirolamo RanieriShinji KawabataGiulia AtzoriShinji TsukamotoGiulia FerrarazzoShinya MatsuzakiGiulia FontanaShiu-Feng HuangGiulia SantoShiv VermaGiuliana CavalloniShohei KawaguchiGiuliana SolinasShoichi FujiiGiulio RossiShuhei YoshidaGiuseppe BronteShun-Fa YangGiuseppe Carlo IorioShunya IkedaGiuseppe Di LorenzoShweta ArasGiuseppe Emmanuele UmanaShweta Pankajkumar LavaniaGiuseppe LoscoccoSidharth MahapatraGiuseppe MorgiaSik Kwan ChanGiuseppe PalmieriSilvana CanevariGiuseppe RivaSilvana MiceliGiuseppe ZimmittiSilvia FilippiGlauco Akelinghton Freire VitielloSilvia GonellaGonzalo VarelaSilvia Martin LluesmaGrażyna Wyszyńska-PawelecSilvia VanniGreg Neil BowdenSilviu Daniel PredaGretchen G. KimmickSimon John Christoph SoerensenGuangjian ZhangSimone MorraGuannan WangSimone SerafiniGuglielmo ManticaSirish DharmapuriGuillaume UlmannSkand ShekharGulisa TurashviliSneha VivekanandhanGulzhanat AimagambetovaSo-Young ChoiGüntülü AkSokol TrunguGuo-Ying WangSoléakhéna KenGuodong FuSoledad García-Gómez-HerasGuowei KimSolveig Roth HoffGwang Ha KimSomnath PandeyH. J. G. Desiree Van Den BongardSonain JamilHacı Mehmet KayiliSonam KumariHaifeng WangSong ParkHaipeng XiaoSonghai ChenHakim MahammediSonja Millert-KalińskaHamid Borghei-RazaviSophia M. SchmitzHans ChristiansenSophie LelorainHans J. M. SmitSoraia MeloHans-Christian KolbergSowjanya ThatikondaHao NieSowmiya A. MoorthieHarikumar RajaguruSoyar SariHaris A. CharalambousSrinadh AnnangiHarleen ChelaStacey A. SantiHarold Yeehau LauStacey J. WinhamHarshpreet ChandokStacy FlowersHaruto NishidaStefan PaepkeHasan MujtabaStefan SajdakHathal HaddadStefania BrozzettiHeather R. MacdonaldStefania CoccoHeleen J. Van BeekhuizenStefania SorrentinoHelen ChappleStefania TroiseHelen J. BulbeckStefanie BomsHelen Jane BoyleStefanie PlageHelmut BeltraminelliStefano BarbiHéloïse BourienStefano DastoliHenrica Maria Johanna WernerStefano FerrettiHenry Hang Fai KwokStefano LuzzagoHermann BussmannStefano MagnoHerwig CerwenkaStefano PaganoHideaki KawabataSteffi M. UrbschatHIdetatsu OtaniStephanie Pei Li SawHildegard Isolde Dietlinde MackStephanie SnowHing Sang Wilfred WongStephen AhnHiro SatoStephen FungHirokazu TakamiStephen KeohaneHiromichi SonodaStephen LiuHiroshi HandaStephen YipHiroyuki DaikoStergios BoussiosHiroyuki NojimaSteven BaumruckerHiroyuki TakahashiSteven BischHiroyuki UetakeStuart PeacockHisaki AibaSu Zy KimHisham FansaSubin Thenkunnel SurendranHo TsoiSubramanyam DasariHon-Yi ShiSudhakar TummalaHongliang HeSukkum ChangHongpeng HeSüleyman EkenHoward H. YangSuman DuttaHoward LimSung-Lang ChenHsin-Hsien YuSunil BadamiHsin-Hung WuSunil Kumar SinghHsu-Heng YenSupriya GuptaHui-Ching ChuangSuresh NarayanasamyHui-Hua HsiaoSusana Sofia MiguelHung-Wei WangSusanne WolferIan S. HagemannSwati PhalkeIan W. TattersallSwee Leong SingIgnacija VlašićSwetha RamadesikanIgor Age KosSyed A. A. RizviIlaria GrassiSyed Azmal AliIlaria GuerrieroSyed N. RahmanIlaria TamagnoSzymon JanczarIlias SkeparniasTadateru MaehataInas FarisTae-Yoon LeeIngrid A. BoereTaha Koray ŞahınIngrid GarajováTai-Li ChenIngrid M. E. DesarTakahiro NishioIoan Mircea PopaTakahiro NoharaIoannis A. KalogiannidisTakahito OhshiroIoannis KyriakidisTakanobu HirosawaIoannis ZachosTakashi HajiroIpshita PrakashTakashi WatanabeIrena IlicTakemi OtsukiIrene Cortés-PérezTakeo YasuIrene FeroceTakeshi KondohIrfan MohamadTaketo YamadaIrina MateiTakumi TakeuchiIris D. NagtegaalTakuya KoieIryna RusanovaTamer AlshafieIsaac RuizTamer Y. SororIsabelle Martel-LafayTania Romo-GonzálezIsao OtsukaTanja Grubić KezeleIsidro MachadoTao FuIvan Shterev DonevTao HuangIvana KholováTao LiIvana SamarzijaTareq SalehIveta SelingerovaTatjana PekmezovicIvonne NelTatsuji MizukamiJacek FurtakTaylor J. ReifJacek R. WilczyńskiTeja Muralidhar KalidindiJacob YoungTengteng YuJacobus Van Der VeldenTeodora Alexa-StratulatJadwiga ManiewskaTeresa BrownJae Hee ChoTeresa PerraJaeyun WangTerry L. NgJaime FornettiTerufumi KawamotoJakub KucharzTessho MaruyamaJames BentleyTheo M. De ReijkeJames ChowTheodoros KoutsandreasJames Janopaul-NaylorTheresia ThalhammerJameson ForsterThiemo DingerJan HrudkaThierry Oscar EdohJan Jens KoltermannThijs J. GiezenJan KorbeckiThomas BurleyJan L. C. LoeffenThomas HankJan Patrick BoströmThomas PabstJan T. LoweryTijl VermassenJan ZivnyTim R. GlowkaJana SmahelovaTimothy AsmisJane LiesveldTimur GürganJane PhillipsTina ŽagarJanice A. BlalockTing Martin MaJaroslav PreslTing-Jung WuJarosław PinkasTingting LiJasminka IgrecTiuri E. KroeseJason PoleTiziana FeolaJason TasoulasTobias OverbeckJason X. ChengTomáš TomášJavad AlizargarTomasz KluzJavier Menéndez-MenéndezTomasz PowrózekJavier Molina-CerrilloTomasz SkrzypekJay DetskyTommaso FarolfiJeanna M. McCuaigTommaso SteccaJeanpierre DrozTomoaki MiyagawaJeffrey BuchsbaumTomoaki YohJehuda SolemanTomoki NakamuraJela BandovicTomoyuki MakinoJennifer CrokeToru IshikawaJennifer LiraToru KannoJennifer M. JohnsonToru SugiyamaJeong-An GimToshiaki TanakaJeonghyun KangToshitsugu FujitaJer-Hao ChangTracey Di SipioJeremy AugustinTraian DumitraşcuJerzy BeltowskiTrevor McGrathJerzy WydmańskiTsuyoshi OhtaJia LiuTze-Chien ChenJiajia XiTze-Kiong ErJiakai HouTzu-Ming ChangJie LeeTzu-Yen HuangJihan XiaUberto Fumagalli RomarioJill Carol RubinsteinUday DeotareJimmy SinglaUenis TannuriJin Hyoung KimUmberto AnceschiJin-Luan LiUmberto MalapelleJin-Tung LiangUpendar GollaJing YuanUros BumbasirevicJingjing ZhangUrsula Maria VoglJingyi ChiValentina CalòJinli HanValentina Elisabetta BounousJiro KikuchiValentine SuteauJo WallerValentyn OksenychJoanna BialekValeria GriecoJoanna Rosak-SzyrockaValeria SebriJoanna ZawitkowskaValerio Di PaolaJoão LeitãoValerio GallottaJoão Miguel CasanovaValerio NardoneJoaquim CarrerasValeriu Marin ȘurlinJoaquim Soares Do BritoVander Monteiro Da ConceiçãoJohan BengzonVanna Chiarion-SileniJohanneke PortieljeVarun BansalJohannes Frank Wilhelmus NijsenVasiliki TzelepiJohannes KloseVaughan ReesJohannes WachVeit RohdeJohn F. NewmanVenkataswarup TiriveedhiJohn F. WardVenkatesh Shankar MadhugiriJohn KildeaVeronica AranJohn L. ReaganVesna Vranić MandušićJohn M. ChirgwinVicente NotarioJohn V. ReynoldsVictor NavaJohn W. BirkVictoria KlepschJoline S. J. LimVictoria L. GreenJonathan NoujaimVictoria TeamJoo Hyun NamViet LeJoon Hyuk ChoiVijay PandeyJordi BrunaVikas YadavJosé Antonio SanchesVikash KansalJosé Daniel SubielaVilas NasareJosé Maria LassoVimoj Janardanan NairJose María RiberaVincent TamJosé Pedraza ChaverriVincent Thomas-de-MontprévilleJosep Maria Serra RenomVincenzo FiorentinoJoseph K. KendalVincenzo QuagliarielloJoseph NassifViola Maria PopovJoseph OjahVirginia CorazziJoshua E. MuscatVirtudes Pérez-JoverJoshua J. X. LiVishnu Muthuraj KumarasamyJosko BozicViswas Raja SolomonJosue Ballesteros-AlvarezVlad PorumbJuan Carlos De VicenteVladimir BedekovićJuan Manuel Mejia-ArangureVladimir K. LyadovJuan Manuel Sepúlveda SánchezVladimir SerezhenkovJulia Furtner-SrajerVui King Vincent-ChongJulian S. RechbergerWael HegazyJulie C. Fanburg-SmithWael TraboulsiJulie EasleyWaldemar WagnerJulie HalletWalentyna BalwierzJulie LapointeWalter ColangeliJulie M. DeleemansWalter H. GotliebJulio-César De La Torre-MonteroWei FengJulius HöhneWei NiJunaid AhmedWei ShaoJung-Ho YoonWei-Chung ShiaJunnliang ChangWei-Hsiung YangJustus BaumgartenWei-Min ChuJyotirmay BiswasWeisi LiuKa Yu TseWeizhen DongKaisa SunelaWen-Tsong HsiehKalra Rajkumar SinghWen-Wei SungKamal Kant SahuWenyi LuoKamil PohlodekWenzhen CaoKamil WdowiakWerner T. W. De RieseKaren A. GelmonWing Lok ChanKaren BräutigamWojciech DudekKaren MulderWojciech M. WysockiKarianne G. FletenWoohyung LeeKarla WillowsXavier X. Sastre-GarauKarol Rawicz-PruszyńskiXiao-An FuKarolina JanionXiaohu HuangKarolina OsowieckaXiaohua WuKatarzyna HojanXiaoli PingKatarzyna TomaszekXiaoxiao SunKathryn E. RoyseXin Shelley WangKatia LeiteXin WangKatie DeCicco-SkinnerXinni SongKatja MohorcicXiuhua WengKatrin ScheinemannXuan LiKatsushi TakebayashiXuan-Mei PiaoKatsuya KobayashiYadollah Abolfathi MomtazKaustuv BhattacharyaYameng LiKazi Abdul MannanYang-Gun SuhKazimierz KuliczkowskiYangyiran XieKazuhiko HashimotoYanhui LuKazuya InokiYanjie ZhangKazuyuki ShimadaYao JiangKeechilat PavithranYaohua YangKelsey L. CorriganYasufumi OmoriKendra L. RatnapradipaYasuhiko SugawaraKengo SasakiYasuhiro OkumuraKenji HayashidaYasushi SasakiKenji YamagataYawei LiKenshirou ShiraishiYfke P. OngenaKensuke MitsunariYi Hui WuKeren LevanonYi LuanKerstin KremeikeYi-Hsuan HsiaoKevin MartellYiğit ÜncüKevin PehrYih Yuan ChenKeyla Vargas-RománYin-Lu DingKhushminder RaiYinglun ZhanKhushwant Singh BhullarYirong ChenKian KaniYoji KishiKinga Hińcza-NowakYolanda López-VidalKirsten LindnerYong Mee ChoKishor PantYongji ZengKjersti FlatmarkYoshihiko KadoyaKlaus WeberYoshikane YamauchiKlaus-Henning KahlYoshinobu HiroseKlazien Matter-WalstraYoshinori InagakiKoichi AndoYoshiyuki SueharaKoji KomoriYosuke MatsuuraKoji MitaYotaro OchiKojiro TauraYoucef M. DerbalKomuraiah MyakalaYoung Wha KohKondapa Naidu BobbaYu Chan ChangKonosuke NakajiYu H. SunKonstantinos ChristopoulosYu KoyamaKonstantinos KatsasYu-Ching LinKoosha PaydaryYu-Ming WangKristaq GazeliYu-Sheng ShenKristen HaaseYue ShengKristjan PaulsonYue ZhaoKrystallenia AlexandrakiYueh-Juen HwuKrzysztof GorynskiYuichiro HatanoKrzysztof SztankeYun-Chen ChangKrzysztof SzyfterYun-Yi ChenKu Youn BaikYurong SongKuang-Chi ChenYuta EndoKuang-Tai KuoYuta MarukiKun WangYuta ShibamotoKun-Yun YehYuto YasudaKyla D. OmilusikYvonne LiKyuwan LeeZachariah FinlyLadislav BatalikZachary LiedermanLaetitia PadovaniZeljko SundovLaffranchi MattiaZesergio MeloLakshmi Vineela NallaZeynep AydiLang QinZheng-Xin WangLashan PeirisZhihan LiuLaszlo TabarZhijian HeLaura AlanizZhiyuan XuLaura Cosima SiegwartZhubo WeiLaura De LellisZhuo HeLaura PatrussiZiqing ChenLaura S. Hiemcke-JiwaZoe ApallaLauren C. HoughtonZoltan HeroldLaurids Østergaard PoulsenZulqurnain SabirLaurie M. ElitZvonko Rumboldt


